# Conserving Freshwater Biodiversity in U. S. Protected Areas – Management Intervention and the RAD Framework

**DOI:** 10.1007/s00267-025-02304-0

**Published:** 2025-12-23

**Authors:** Kellie J. Carim, Hannah Adkins, Remi Murdoch, Leah Simantel, Andrea Stephens, Matthew Webster, Kira Hefty, Lisa A. Eby

**Affiliations:** 1 Aldo Leopold Wilderness Research Institute, Rocky Mountain Research Station, U.S. Forest Service, Missoula, MT USA; 2 Environmental Studies Program, College of Humanities and Sciences, University of Montana, Missoula, MT USA; 3 Wildlife Biology Program, W.A. Franke College of Forestry and Conservation, University of Montana, Missoula, MT USA; 4 The Wilderness Institute, W.A. Franke College of Forestry and Conservation, University of Montana, Missoula, MT USA

**Keywords:** Wilderness, Wilderness character, Resist-Accept-Direct, Fish stocking

## Abstract

Protected areas are considered a critical antidote to global biodiversity loss. Yet, protected areas have not effectively preserved freshwater biodiversity compared to other taxonomic groups, in part because they are not designed or managed with freshwater ecosystems in mind. Additionally, historical and current human activities have degraded freshwater biodiversity, and climate driven transformation constrains the ability of protected areas to preserve freshwater biodiversity into the future. Management intervention is an important tool to ensure protected areas support current and future ecological, socio-cultural, and economic values surrounding freshwater biodiversity. Yet applying interventions is challenging because many protected areas limit human manipulation and control. The Resist-Accept-Direct (RAD) framework is a tool for weighing management approaches in an expanded decision space. As such, the RAD framework may help identify the most appropriate approaches to address ecological transformation while balancing the mandates and values of a given protected area. In this paper, we review federally protected areas of the United States and their varied ability to support freshwater biodiversity. We then recast past management approaches through a RAD lens, examining how they *resist*, *accept*, or *direct* freshwater biodiversity loss in these protected areas. This illustrates how elements of the RAD framework are already being applied and provides a foundation for managers to more formally *resist*, *accept*, or *direct* freshwater biodiversity loss in protected areas moving forward. We conclude with considerations for applying the RAD framework at the intersection of freshwater biodiversity and protected areas to benefit values of current and future generations.

## Introduction

Biodiversity loss is a global conservation concern, with an estimated 69% decline in wildlife biodiversity world-wide since 1970 (World Wildlife Fund 2022). Protected areas are considered a critical antidote to global biodiversity declines (Dinerstein et al. 2019). These areas are typically managed to limit human activities and other threats (e.g., invasive species) that negatively affect wildlife habitat, ecosystem health, ecosystem services, and cultural values for the benefit of current and future generations (Dudley and Stolton 2008). Evidence shows that protected areas do generally support greater biodiversity relative to non-protected areas (Gray et al. 2016); however, these benefits vary substantially by taxonomic group (Brodie et al. 2023). In freshwater ecosystems, biodiversity has declined globally by 84% since 1970 (World Wildlife Fund 2022). This is more than double the rates for marine and terrestrial ecosystems (World Wildlife Fund 2018, Reid et al. 2019), and suggests that the mere existence of protected areas is not providing sufficient benefits to freshwater taxa (Acreman et al. 2020).

The disproportionate declines of freshwater biodiversity have been attributed to the fact that most protected areas are not designated or managed with freshwater systems in mind (Saunders et al. 2002, Cowx and Aya 2011, Finlayson et al. 2018, Acreman et al. 2020). Additionally, protected areas with specific goals for conserving freshwater ecosystems often lack a comprehensive focus on biodiversity and do not protect the larger hydrologic and geomorphic processes necessary for freshwater systems to function (Rothlisberger et al. 2017, Miqueleiz et al. 2023, Valentim et al. 2024). To address these conservation gaps, aquatic ecologists have made recommendations for management of terrestrial protected areas to increase benefits to freshwater systems and for designation of new protected areas specific to freshwater systems. Broadly, these recommendations include: prioritizing protections at a drainage scale, connecting and maintaining freshwater corridors, supporting natural flow regimes, addressing habitat and water quality issues and protecting riparian habitats, suppression and removal of nonnative/invasive species, incorporating socio-cultural and economic values into management decisions, and conducting monitoring and research to evaluate management success and inform adaptive strategies (Cowx and Aya 2011, Saunders et al. 2002, Finlayson et al. 2018, Acreman et al. 2020; Dudgeon and Strayer 2025).

**Fig. 1:**
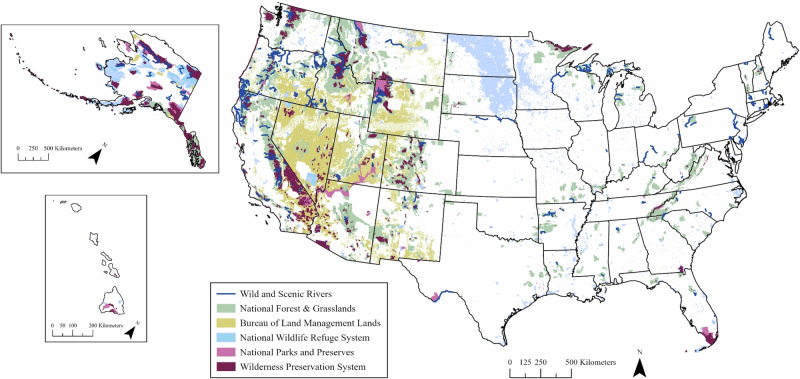
Map of federally designated protected areas in the United States with the greatest potential to benefit freshwater biodiversity. The protected areas shown are those commonly managed for natural character and biodiversity at the national level. Note that this map excludes designations managed by the National Parks Service whose management is unrelated to ecological or biodiversity conservation (e.g., National Battlefields, National Parkways, National Historic Sites, etc.). See Table S1 for data sources used to produce this map

In the United States, there are a variety of federally protected areas, each with varying potential to incorporate these recommendations for freshwater biodiversity conservation (Fig. 1, Table 1). This potential in part depends on the purpose and legal mandates associated with each protected area type (described below in Section I). For example, many of the recommendations above require management interventions to address sources of degradation and current threats to freshwater biodiversity. However, mandates that explicitly limit human manipulation and control in some protected areas can make it difficult to implement management interventions, even if those interventions would benefit ecological form and function (Kelly and Landres 2023). Additionally, climate-driven transformations across this protected area network have further altered aquatic systems and their biological communities away from historical baselines (Milly et al. 2008), leading to increased variability and novel conditions (e.g., Asadieh and Krakauer 2017). Furthermore, ensuring that management decisions meet socio-cultural and economic values can be difficult as these also change over time (see Holmes 2022). As baselines and values inevitably shift, the feasibility and efficacy of management approaches to meet conservation targets become increasingly uncertain (Schuurman et al. 2020). A conceptual framework that assists managers in incorporating the above recommendations under these challenging circumstances could increase the long-term success of management efforts across differentially protected areas.

**Table 1:** Federally designated protected areas of the United States with the greatest potential for freshwater biodiversity conservation

Protected Area		Administering Agency	Description	Total area designated	Area designated as Wilderness (% of total)	Relevant legislation
Wild and Scenic Rivers		BLM, USFS, FWS, NPS	Wild and Scenic Rivers are segments of rivers with unique “outstandingly remarkable values” designated to preserve their free-flowing nature. Outstandingly remarkable values are categorized as scenic, recreational, geologic, fish and wildlife, historical, or cultural. There are three categories of designations including “wild,” “scenic,” and “recreational.” “Wild” designations are the most remote (un-roaded) and are generally inaccessible except by trail. “Scenic” and “Recreational” designations represent categories of increasing development along shorelines, accessibility by road, and may have previously supported water impoundments or diversions. Wild and Scenic Rivers may be administered by USFS, BLM, NPS, or FWS.	21,673 river km^**a**^	10,384 river km designated as “wild” (48%)	Wild and Scenic Rivers Act of 1968 (P.L. 90-542)
National Forests and Grasslands		USFS	National Forests and Grasslands are considered “multi-use,” and are managed to support habitat for fish and wildlife, along with recreation, land use, and natural resource extraction. National Forests and Grasslands may contain federally designated wilderness.	780,949 km^2 **b**^	148,399 km^2^ (19%)	National Forest Management Act of 1976 (P.L. 94-588)
BLM Managed Lands (including National Conservation Lands)		BLM	Lands managed by the BLM are considered “multi-use,” and include land use and natural resource extraction. Although fish and wildlife conservation occurs on all BLM lands, national conservation lands are a relatively new category of lands specifically managed for non-extractive uses, including preservation of cultural, ecological, and scientific values. National conservation lands may contain federally designated wilderness.	988,965 km^2^ (National Conservation Lands 101,993 km^2^)^**c**^	41,156 km^2^ (0.4% of total; 40% of National Conservation Lands)	Federal Lands Policy and Management Act of 1976 (P.L. 94-579), National Conservation Lands Act of 2009 (P.L. 111-11)
National Wildlife Refuges (including Waterfowl Protection Areas)	FWS		National wildlife refuges are a network of lands and water administered by the FWS for the conservation, management, and restoration of fish, wildlife, and plant resources and their habitats. Refuges may contain federally designated wilderness areas.	3,464,594 km^2^ (approx. 3,075,611 marine)^**d**^	83,781 km^2^ (2%)	National Wildlife Refuge System Administration Act of 1966 (P.L. 89-669); National Wildlife Refuge System Improvement Act of 1997 (P.L. 105-57)
National Parks		NPS	National parks are lands set aside to preserve the United States’ natural and cultural resources. National parks may contain federally designated wilderness areas; those without wilderness are still typically managed to protect wilderness character.	319,242 km^2**e**^	179,463 km^2^ (56%)	Organic Act of 1916 (39 Stat.535, 16 U.S.C. 1.)
Wilderness Areas		BLM, USFS, FWS, NPS	Wilderness Areas are managed to preserve five primary wilderness characters including the undeveloped character, untrammeled character, opportunities for unconfined recreation and solitude, natural character, and “other” characters (i.e., geological and historical values, and opportunities for science and education; see Landres et al. 2015). Wilderness Areas may be administered by USFS, BLM, NPS, or FWS.	452,799 km^2**f**^	452,799 km^2^ (100%)	Wilderness Act of 1964 (P.L. 88-577)

^**a**^ www.rivers.gov/numbers

^**b**^ https://www.fs.usda.gov/land/staff/lar/LAR2012/LAR_Book_FY2012_A4.pdf

^**c**^ https://www.blm.gov/sites/default/files/docs/2021-08/PublicLandStatistics2020_1.pdf

^**d**^ https://www.fws.gov/sites/default/files/documents/2021-annual-report-of-lands-with-data-tables_2.pdf

^**e**^ https://www.nps.gov/subjects/lwcf/acreagereports.htm

^**f**^ www.wilderness.net

The protected areas listed are those commonly managed for natural character and biodiversity at the national level, as detailed under “Description”. Abbreviations under “Administering Agency” are as follows: BLM- Bureau of Land Management; USFS- U. S. Forest Service; FWS- U.S. Fish and Wildlife Service; NPS - National Park Service. Under “Total Area Designated”, metrics exclude administrative units and designations that lack focus on preservation of natural character or biodiversity (e.g., national memorials, parkways, monuments, etc.)

The Resist-Accept-Direct (RAD) framework was recently introduced to inform management decisions in the context of inevitable ecological transformation, defined as change in an ecosystem that differs dramatically and irreversibly from prior structure and function (Schuurman et al. 2020, Lynch et al. 2021a, Thompson et al. 2021). The RAD framework assists managers in balancing dynamic ecological, socio-cultural, and economic values by providing three pathways for addressing ecological transformation: *resist*, *accept*, or *direct*. Lynch et al. (2021a) defines these as:

*Resist* - maintaining current or historical ecosystem structure and function through management intervention.

*Accept*- avoiding any management intervention and accepting the ecosystem structure and function that emerge from the ecological transformation.

*Direct-* accepting that ecological transformation is occurring but intervening to steer the transformation towards an ecosystem state with a particular structure and function.

Actions that *resist* transformation aim to maintain the ecological system within a current or historical range of variability. In contrast, *accepting* and *directing* ecological transformation will result in conditions that differ from current or historical baselines (Schuurman et al. 2022; Moss et al. 2025).

**Fig. 2:**
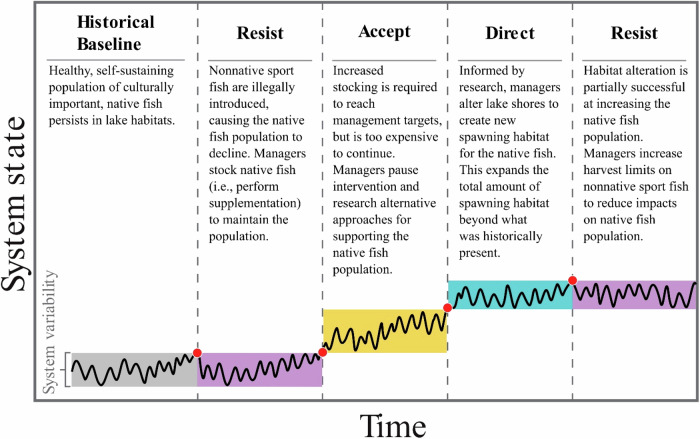
A simplified, hypothetical series of responses to loss of freshwater biodiversity in a protected area, overlaid with a heuristic pathway (modified from Lynch et al. 2021b) that combines RAD with adaptive management. Shaded zones represent the range variability for the historical baseline (gray) or acceptable range of variability for the chosen management approach- *resist* (purple), *accept* (yellow) or *direct* (teal). When the observed state of the system (black line) exceeds the acceptable range of variability for a given approach (denoted by red dots), managers may choose to reevaluate values and adjust their management approach

The RAD framework is conceptual process to help managers identify priority values and consider a broader range of management approaches with ecological uncertainty in mind (Schuurman et al. 2025, Sesser et al. 2025). Combining the RAD framework with step-by-step decision-making guides aids managers in selecting the approach that best meets conservation and management objectives (Sesser et al. 2025). For example, combining the RAD framework with an adaptive management framework promotes an iterative process of monitoring and evaluation to determine when one approach may no longer be feasible, and provides a pathway to pivot to an alternative approach (Fig. 2; Lynch et al. 2021b, Cravens et al. 2024, Sesser et al. 2025). The RAD framework is beginning to be applied in freshwater systems (Rahel and Lynch 2022) but has sparingly been used to inform management decisions in protected areas with conflicting mandates. By supporting an expanded decision space to weigh management options, the RAD framework can assist managers working to balance ecological uncertainty, freshwater biodiversity loss, and conflicting mandates in protected areas.

In this paper, we examine management approaches that deliberately *resist*, *accept*, or *direct* transformation of freshwater biodiversity in protected areas of the United States. We begin with a description of the types of protected areas and their potential to benefit freshwater biodiversity (Section I), followed by a brief review of the human activities that have degraded freshwater biodiversity in these protected areas (Section II). We then examine how previous approaches to managing freshwater biodiversity in protected areas fit within the scope of the RAD framework (Section III). Examining past management approaches through a RAD lens demonstrates how managers of protected areas and freshwater systems already engage with various elements of the framework. Furthermore, this examination provides context that supports decisions to intentionally *accept* freshwater biodiversity loss in protected areas or to intervene when doing so would provide the greatest benefit to the ecological and social-cultural values of a protected area. We end with a discussion on additional considerations for applying the RAD framework and broader opportunities for advancing freshwater biodiversity across protected areas in the United States (Section IV).

## Section I: Protected Areas of the United States and Potential to Benefit Freshwater Biodiversity

There are six types of protected areas in the United States with potential to support freshwater biodiversity (Table 1). The ability of each type to benefit freshwater biodiversity varies based on the purpose and legal mandates for each designation and is detailed below. We begin with a description of the only protected area type to focus on freshwater systems, followed by land-based protected areas listed in relative order of protection level and human activity (see Dudley and Stolton 2008).

### Wild and Scenic Rivers

The Wild and Scenic Rivers Act of 1968 (P.L. 90-542) created the first ever national river conservation designation (Valentim et al. 2024). This act protects segments of free-flowing rivers with “outstanding remarkable values,” defined as the combination of “scenic, recreational, geologic, fish and wildlife, historic, cultural, or other.” Protected river segments are categorized as “recreational”, “scenic”, or “wild” based on the amount of human development along shorelines and presence of previous impoundments. The act protects over 21,500 river km across the United States (Fig. 1), with the median designation length of 48.8 river km (https://rivers.gov/maps-graphics). While emphasis on protecting free-flowing rivers is beneficial to many biotic and abiotic processes of freshwater systems, the broader structure of wild and scenic river designations poses several limitations to protecting freshwater biodiversity. For example, “fish” is not an outstanding remarkable value for all designations, and no single outstanding remarkable value directly protects freshwater biodiversity at a community level. Furthermore, because the designation focuses on segments of mainstem rivers rather than whole river drainages or watersheds, the hydrologic and geological processes necessary for healthy stream and river habitats are not fully protected (Rothlisberger et al. 2017; see also Poff et al. 1997). Despite these limitations, wild and scenic rivers remain the only federal designation in the United States that focuses specifically on protecting freshwater systems.

### National Forests and Grasslands and the Bureau of Land Management

National Forests and Grasslands (managed by the U.S. Forest Service) and lands managed by the Bureau of Land Management are considered “working lands” that balance ecological values with multiple other uses including recreation, land use, and natural resource extraction (National Forest Management Act of 1976, P.L. 94-588, Federal Lands Policy and Management Act of 1976, P.L. 94-579). Combined, these two protected area types represent approximately 72% of all protected land area in the United States (Table 1) and often encompass multiple connected river subbasins (defined as 8-digit hydrologic unit; see https://nas.er.usgs.gov/hucs.aspx), resulting in a high potential to provide watershed-scale protections for freshwater systems. These protected area types are distributed nationwide but occupy the greatest land area in the western coterminous United States (Fig. 1). While managing for multiple uses is an important economic value, historical and ongoing activities have degraded freshwater habitats in portions of these protected areas, contributing to freshwater biodiversity declines (Jones et al. 1999, Kershner et al. 2004, Herbst et al. 2012, Roper et al. 2018).

### National Wildlife Refuges and National Parks

The National Wildlife Refuge System, comprised of wildlife refuges managed by the U.S. Fish and Wildlife Service, has a more deliberate focus on biodiversity relative to other protected area types, with a mandate to prioritize conservation of fish, wildlife, plants and their habitats (National Wildlife Refuge System Improvement Act of 1997, P.L. 105-57). The three largest wildlife refuges (all located in Alaska) each exceed 34,000 km^2^ and encompass one or more whole river subbasins. However, the median wildlife refuge size (excluding waterfowl protection areas) is under 33 km^2^ (https://gis-fws.opendata.arcgis.com/datasets/9c49bd03b8dc4b9188a8c84062792cff/explore; retrieved August 12, 2024) and refuges are not evenly distributed across ecoregions (Fig. 1; Scott et al. 2004). Waterfowl protection areas are numerous but small disconnected units within the National Wildlife Refuge System (more than 3000 units, median size = 0.65 km^2^) located primarily in the upper Midwest that protect breeding habitat for migratory waterfowl (Migratory Bird Hunting and Conservation Stamp Act 16 U.S.C. 718–718 k). The emphasis of wildlife refuges on protected focal taxonomic groups has supported the persistence of endemic species, including freshwater fish and invertebrates (e.g., Brown 2021, Johnson et al. 2022). However, the size and distribution of wildlife refuges is a limiting factor; while these units certainly benefit freshwater biota, they are highly fragmented and not managed explicitly to protect freshwater biodiversity or the function of freshwater ecosystems.

National parks, managed by the National Park Service, take broader management focus aimed at protecting “natural and cultural resources” balanced with public visitation (Organic Act of 1916 39 Stat.535 16 U.S.C. 1, National Park Service 2017). Although national parks tend to be larger by unit than wildlife refuges (median size = 890 km^2^, www.nps.gov/subjects/lwcf/acreagereports.htm, retrieved November 18, 2024), they too rarely encompass entire watersheds. National parks are generally well distributed across ecoregions of the U.S., which increases their potential to represent biodiversity at a national scale (Fig. 1; Lawrence et al. 2011). Not surprisingly, over 60% of fish and amphibian species native to the United States are represented across the national park system, although species of conservation concern are underrepresented relative to unimperiled species (Lawrence et al. 2011, LaFrance et al. 2024). The connection between visitation and biodiversity in national parks can be mutualistic as visitors may be more attracted to parks with greater biodiversity (e.g., Siikamäki et al. 2015). Additionally, national parks have a long history of applied conservation research (Vukomanovic and Randall 2021), which has supported exploration of innovative approaches to conserving freshwater biodiversity (e.g., Galloway et al. 2016).

Both national parks and national wildlife refuges typically do not permit land use or resource extraction and actively promote habitat restoration activities to address human impacts, which generally results in higher quality habitat (Scott et al. 2004, Meretsky et al. 2006, Goetz et al. 2009, Zellmer 2014), and therefore greater potential to benefit freshwater biodiversity.

### Federally Designated Wilderness Areas

The National Wilderness Preservation System represents over 800 federally designated wilderness areas across the United States. These land designations may be nested within any of the terrestrial protected areas listed above (see Table 1) and are managed to preserve their “primeval” and “natural” characters and to provide recreational opportunities, while minimizing the effects of modern human activities (Wilderness Act of 1964, P.L. 88-577, see also Landres et al. 2015). Note that these same mandates generally apply to national parks and wildlife refuges, as even non-wilderness portions of these protected areas tend to be managed with wilderness characters in mind (Landres et al. 1998). While most wilderness areas are small (median size = 91.5 km^2^; https://wilderness.net/visit-wilderness/gis-gps.php) and do not encompass entire watersheds, they commonly span headwater areas. As a result, the benefits of many wilderness areas to freshwater biodiversity and ecosystem function likely extend beyond their boundaries to downstream portions of the watershed (see Colvin et al. 2019, Brown et al.^2016^). Of all protected areas in the United States, federally designated wilderness areas have the highest restrictions on human activities and influence. Yet, wilderness areas are not necessarily pristine- legacy effects from human activities prior to designation (such as introduction of nonnative species, mining, and dams) persist in many wilderness areas (Hubbard et al. 2000, Landres et al. 2001, Zellmer 2012; see also Section III). This creates challenges for managers as mandates to limit human influence are at odds with management activities to restore the quality, function, and biodiversity of freshwater systems in wilderness areas (Cole and Yung 2012).

### Limitations and Strengths for Protected Areas as a Network

Beyond the limitations of each protected area type, all are hindered in their efficacy to protect freshwater biodiversity because they may not protect the most biodiverse habitats or ecosystems (Panlasigui et al. 2018), are limited in mitigating negative factors originating outside of protected area boundaries (Landres et al. 1998, Goetz et al. 2009, Wade et al. 2011), and may not fulfill all habitat requirements for freshwater biota, particularly for migratory species (Bower et al. 2015). In many instances, these broader limitations could be addressed with coordinated efforts across protected areas, particularly in the western United States where protected areas cover much of the landscape and often share boundaries (Fig. 1). Such efforts could maintain and restore natural flow regimes (Dudgeon et al. 2006), create corridors for migratory species, and facilitate access to areas that remain buffered from climate driven transformation (e.g., Isaak and Young 2023).

Yet even with coordinated efforts across the protected area network, localized interventions within individual units are critical for addressing threats and ensuring persistence of freshwater biodiversity (Cowx and Aya 2011, Acreman et al. 2020, Dudgeon and Strayer 2025). Such interventions may be easier to apply in multi-use protected areas where human activities are less restricted, but are scrutinized in the protected areas with mandates to limit human activities that manipulate or “trammel” the ecological system (Kelly and Landres 2023). For the remainder of this paper, we focus on protected areas designated as wild and scenic rivers, national wildlife refuges, national parks, and wilderness. We focus on these designations because, relative to multi-use protected areas, they more often face the dilemma between management interventions to protect biodiversity versus limiting human influence.

## Section II: Freshwater Biodiversity in the United States: From Extraction to Protection

For over 200 years, human activities that modify aquatic communities and natural processes of freshwater systems have occurred in areas of the United States now designated as protected (Matthews et al. 1985, Squillace 2014, Loomis 2017). Although activities such as logging, mining, and livestock grazing are not necessarily centered on aquatic resource use, they have negatively influenced aquatic habitat and biota by diverting water, altering hydrogeomorphic processes, and degrading water quality (Jones et al. 1999, Kershner et al. 2004, Herbst et al. 2012, Giam et al. 2018, Roper et al. 2018). Additionally, unsustainable trapping of North American beavers (*Castor canadensis*) and subsequent declines of beaver dams have altered water and sediment storage across watersheds (Wohl 2021, Scamardo et al. 2022), and are associated with declines of other aquatic species (Hossack et al. 2015). Many of these activities no longer occur in protected areas, with some exceptions (Cole and Landres 1996, Squillace 2014), yet their negative effects are still apparent decades later (Meretsky et al. 2006, Squillace 2014).

Human activities that focus specifically on aquatic resource use have also had legacy effects on freshwater systems in protected areas. For example, hundreds of dams built for stream flow regulation and water storage persist in areas now designated as wilderness (Zellmer 2012). Efforts have been made in some wilderness areas to remove these structures and restore natural flow regimes (e.g., National Park Service 2009, U.S. Forest Service 2024), but most remain even if they are no longer functional (Zellmer 2012). Perhaps the most notable and broad ranging human alteration of aquatic systems across U.S. protected areas is fish stocking. To promote recreational fishing opportunities, translocation of fish, particularly nonnative trout, has occurred across public lands since the early 1870s, including in systems that were historically fishless (Halverson 2010). In many locations, fish stocking activities that preceded the designation of the protected areas were allowed to continue after designation as a “pre-existing use”.

Although intended to benefit socio-cultural and economic values, fish stocking has dramatically altered the ecological form and function of freshwater ecosystems (Table S2). Detriments to freshwater biodiversity range from genetic repercussions for individual populations to alteration of ecosystem level processes (Holmlund and Hammer 2004, Eby et al. 2006, Cucherousset and Olden 2011, Epifanio and Waples 2016), and can extend beyond the geographic area where stocking occurred (Dunham et al. 2004). These negative effects are pronounced when stocking nonnative fish and in waterbodies that were historically fishless, although negative effects may still occur when stocking strains of native species (e.g., introduction of novel pathogens, outbreeding depression; see Table S2).

While fishing continues to be a popular recreational pastime in the United States (U.S. Fish and Wildlife Service 2016), socio-cultural values have shifted in recent decades from a focus on recreational opportunities towards conservation of native species and biodiversity (Rahel 2016, Chiapella et al. 2018, Arlinghaus et al. 2021). Today, the federal agencies that administer wilderness and other protected areas take varied approaches to fish stocking based on their mission and whether recreational use of the protected area is a priority (Landres et al. 2001, Association of Fish and Wildlife Agencies 2006). Stocking for recreational opportunities in many protected areas has declined relative to historic rates (Pister 2001) and is usually limited to waterbodies with populations established prior to designation (Association of Fish and Wildlife Agecies 2006). In some protected areas, management for recreational fishing opportunities has refocused efforts towards stocking species that are regionally native (Bonneville Power Administration 2006, U.S. Forest Service 2006). In other areas, efforts have been made to remove stocked fish entirely and restore systems to their historically fishless state (see Section III).

Management interventions that counteract the negative effects of human activities (beyond fish stocking) on freshwater biodiversity have also increased in recent decades (e.g., Bash and Ryan 2002, Sudduth et al. 2007). However, performing these interventions remains challenging in highly protected areas. For example, removing defunct dams or adding large woody debris to streams are increasingly common management practices to restore freshwater habitat and hydrologic function (O’Connor et al. 2015, Roni et al. 2015). While these interventions in support the “natural” character of protected areas, they require the use of mechanized equipment and may be considered “trammeling”- both of which are restricted under the Wilderness Act of 1964.

Furthermore, climate driven transformations will alter the ability of protected areas to support their historical biological communities (Parks et al. 2023). For example, increasing water temperatures are predicted to extirpate cool and cold-water fish species from a large proportion of their home ranges over the next century, with negative consequences for socio-cultural and economic values (Jones et al. 2013). Yet even monitoring temperature-related changes to freshwater habitat without intervention typically requires installation of equipment (temperature sensors), which is also restricted in many protected areas. Thus, managers of freshwater biodiversity will be challenged by conflicting mandates even in protected areas with little to no history of extractive use or manipulation.

## Section III: Recasting Past Conservation Interventions into the RAD Framework

In many protected areas, mandates to minimize human influence often promote management approaches that *accept* biodiversity loss (Kelly and Landres 2023). As socio-cultural values and legal mandates have shifted towards a focus on conservation in the later 20th century, actions that *resist* ecological change in protected areas have become common (Schuurman et al. 2022, Lieberman et al. 2018). Yet, the efficacy of interventions to *resist* biodiversity loss can be uncertain (Detmer et al. 2025). Furthermore, these interventions may become ineffective under future climate conditions, putting the relevance of protected areas into question. For example, research suggests that Joshua trees (*Yucca brevifolia*) may be extirpated from Joshua Tree National Park by the end of the 21^st^ century due to changes in temperature and annual precipitation (Sweet et al. 2019). To maintain the relevance of protected areas to society, managers are increasingly faced with difficult decisions to ensure that protected areas continue to serve the ecological and socio-cultural values that guided their designation (Kelly and Landres 2023).

The RAD framework can assist in these circumstances by encouraging managers to consider a broader suite of approaches that support socio-cultural, economic, and ecological values of a protected area, rather than resorting to traditional management defaults (Schuurman et al. 2020). Below we highlight a variety of common actions that may be taken to *resist*, *accept*, or *direct* freshwater biodiversity loss in protected areas (see also Table 2). The RAD framework was not directly applied when making the management decisions described below. However, these examples illustrate how managers are already integrating elements of the framework when addressing the unique challenges of managing freshwater biodiversity in protected areas. This sets precedents for managers applying the framework to intentionally *accept* freshwater biodiversity loss or intervene when doing so would provide the greatest benefit to the values of a protected area.

**Table 2:** Examples of management activities that may occur under approaches that *resist*, *accept*, or *direct* freshwater biodiversity loss

Management Approach	Example Management Activity
Resist	Supplementation to increase population densities or to address loss of genetic diversity
	Reintroduction of species to areas where they have been extirpated
	Habitat alteration or restoration to improve habitat conditions for native freshwater species
	Suppression or eradication of introduced species that negatively affect aquatic biodiversity and ecosystem function
Accept	Research, inventory, and monitoring to better understand ecological transformation and to use protected areas as an experimental control
	Outreach and education on freshwater biodiversity loss, using protected areas as an example
Direct	Translocation of species outside their historical range to support their persistence in a given protected area, or to support socio-cultural and/or economic values
	Modification of habitat away from historical conditions to expand habitat for freshwater species of interest

Note that some activities may fall under more than one RAD management approach depending on the context of a specific management objective and protected area

### Accepting Freshwater Biodiversity Loss in Protected Areas

In protected areas, the choice to *accept* loss of freshwater biodiversity caused by direct and indirect effects of human activities may stem from a tendency to favor restraint of human interventions over ecological and socio-cultural values (Stephenson and Millar 2014), from lack of available resources (e.g., staff, supplies and equipment, funding), or from a paucity of information to support a different approach (Clifford et al. 2021). *Accepting* biodiversity loss is considered a default strategy when the action is taken without deliberate consideration for *resisting* or *directing* actions (Thompson et al. 2021). In these instances, the choice to *accept* is rarely documented, and it is therefore difficult to find many explicit examples in protected areas. One example of *accept* may be allowing self-sustaining populations of introduced fish to persist in protected areas following cessation of active stocking efforts (e.g., Wiley 2003). This choice may degrade ecological values by perpetuating one or more of the negative outcomes outlined in Table S2. However, it may also balance socio-cultural and economic values by maintaining recreational fishing opportunities, while avoiding the financial costs and human influence required for continued stocking or eradication efforts. Managers may also intentionally *accept* ecological transformation if they seek to learn about changing ecological conditions or for use in a “portfolio approach” alongside other management approaches (Aplet and Mckinley 2017, Magness et al. 2022). When combined with research, inventory, and monitoring, *accepting* the ecological transformation can provide an opportunity to better understand the system undergoing change, and inform future interventions that *resist* or *direct* biodiversity loss through adaptive management (Fig. 2; Lynch et al. 2021b). In this context, protected areas may be used as “study controls” when evaluating the effectiveness of interventions in other locations where *resisting* and *directing* actions are more feasible. This approach may be more appropriate for protected areas where intervention is less palatable (see Clifford et al. 2021). Finally, *accepting* biodiversity loss may be the only choice in circumstances where ecological change has altered the system to a point that it can no longer support the desired ecological state (e.g., Stubbington et al. 2024; see also Box 1).

Within the RAD framework, decisions that *accept* biodiversity loss are those that adequately incorporate ecological and socio-cultural values, and weigh the resource needs and likelihood of success associated with alternative actions. If we expect protected areas to serve as refuges for biodiversity, it may also be important to consider how *accepting* loss within protected areas affects biodiversity and ecosystem function in adjacent landscapes (beyond protected area boundaries).

Box 1 Accept- Loss of Lake Trout in the Boundary Waters Canoe Area and Surrounding Region The northeast region of Minnesota is home to thousands of cold- and cool-water lakes renowned for their recreational fishing opportunities. As reported in 2002, this region supported over 35% of the state’s cold-water angler trips, contributing significantly to the state’s > $140 million cold-water angling industry (Gartner et al. 2002). Not surprisingly, the region has a long history of fish stocking for recreational use, with records dating back to the 1900s (Minnesota Department of Natural Resources & U.S. Forest Service 2020). Lake trout (*Salvelinus namaycush*) are a regionally iconic fish native to a number of cold-water lakes in the region, including the Boundary Waters Canoe Area Wilderness (BWCAW), located on the Superior National Forest (Underhill 1989).

East Bearskin Lake, located partially within the BWCAW, historically supported a relatively simple fish community where lake trout was the top predator. Fish stocking in East Bearskin and other lakes in northeastern Minnesota throughout the early and mid-20th century introduced other regionally native predatory species, such as walleye (*Sander vitreus*) and smallmouth bass (*Micropterus dolomieu*). In many waterbodies, introductions negatively affected native lake trout populations, and may have aided the extirpation of lake trout in East Bearskin Lake by the 1950s (Minnesota Department of Natural Resources 2023).

To restore native lake trout populations and support a recreational fishery, the Minnesota Department of Natural Resources (MN DNR) reintroduced lake trout to East Bearskin Lake in 1993, with subsequent stocking in odd numbered years from 1997 – 2021 (Minnesota Department of Natural Resources 2023). Early efforts to reestablish lake trout in East Bearskin were promising, with evidence of survival and rapid growth of stocked fish in surveys conducted in 1998 (Minnesota Department of Natural Resources 1998). However, no evidence of natural reproduction was observed in later surveys, and few lake trout survived to catchable size (Minnesota Department of Natural Resources 2023). Beginning in the early 2000s, survival of stocked lake trout declined substantially in East Bearskin Lake (Minnesota Department of Natural Resources 2023; E.J. Isaac, MN DNR, pers. comm). The failure of stocked lake trout to survive in East Bearskin is not fully understood. However, lake trout declines in relatively shallow lakes throughout their native range have been attributed to climate related increases in lake temperatures that reduce the availability of suitably cold and oxygenated habitat during warmer summer months (Jacobson et al. 2010, Minnesota Department of Natural Resrources 2023). In 2022, the MN DNR discontinued stocking of lake trout throughout northeastern Minnesota due to mixed success. Surveys in some lakes demonstrated stocking had been successful at establishing a self-sustaining fishery and was no longer necessary (M. Weberg pers. comm.). In other lakes where stocking efforts failed to restore or augment lake trout populations, (like East Bearskin Lake), cessation of stocking may lead to population declines or even extirpation. However, *accepting* these declines may be temporary. It is possible that other strains of lake trout may have higher survival and spawning success in these lakes. If such strains could be identified and propagated, they may be used to support future conservation stocking efforts in the region.

The status of lake trout populations in other lakes throughout the BWCAW is uncertain due to challenges associated with managing in a wilderness context. Population and habitat surveys for lake trout are needed to fully understand changes to recreational fishing opportunities, assess declines in the “natural” character of the BWCAW, and to inform efficacy of possible management interventions. For example, a memorandum of understanding between the U.S. Forest Service and MN DNR permits stocking of regionally native fish by the MN DNR in 111 lakes within the BWCAW (Minnesota Department of Natural Reserouces & U.S. Forest Service 2020), but information is needed to determine whether stocking to maintain or restore lake trout populations would be ecologically warranted and successful. Collecting this information is challenging because the tools necessary to conduct robust surveys, such as mechanized equipment (e.g., motorboats) and temporary installations (e.g., temperature sensors) would diminish the “undeveloped” character of this wilderness area. Additionally, broader conversations among fisheries and wilderness managers, rights holders (i.e., local Native American Tribes), and the public would likely be necessary to determine whether conservation stocking of lake trout within the BWCAW would support both ecological and socio-cultural values of the protected area. BWCAW managers and MN DNR fisheries managers are continually working together to balance management goals as well as the suite of values associated with this wilderness area.

### Resisting Freshwater Biodiversity Loss in Protected Areas

When the effects of biodiversity loss on ecological, socio-cultural, and economic values are deemed too great to *accept*, managers may choose to *resist* ecological transformation. This decision may be motivated by placing greater value on retaining the historical ecological conditions of a protected area, or by legal mandates, such as management of species listed under the Endangered Species Act of 1973 (P.L. 93-205). In other instances, they may reflect values focused on preserving culturally relevant species for future generations (e.g., Shultz et al. 2022). *Resisting* actions are diverse and may address issues ranging from genetic integrity of a population to hydrologic function of entire watersheds (see below). Common *resisting* actions in freshwater systems include supplementation of populations, reintroduction of extirpated species, habitat alterations, and eradication of introduced/invasive species.

Supplementation and reintroduction are examples of *resisting* actions that aim to augment or reestablish populations of native species. Supplementation is defined as the addition of individuals to a population. This approach may be used to counteract the negative effects of inbreeding (Whiteley et al. 2015). To our knowledge, there are no documented instances of genetic rescue for freshwater species in protected areas of the United States, although this type of intervention has been implemented in protected areas (including wilderness) for other taxa (e.g., Hogg et al. 2006, National Park Service 2018). Supplementation has also been used to increase population densities of declining freshwater species. For example, captive-reared Ozark hellbender salamanders (*Cryptobranchus alleganiensis bishop*) have been used to augment federally Endangered populations of the White River Basin (U.S. Fish and Wildlife Service 2011a, Ettling et al. 2013, Ettling et al. 2017), which includes the Devil’s Backbone Wilderness, Irish Wilderness, and Eleven Point Wild and Scenic River in Missouri. In comparison, reintroduction efforts, defined as the introduction of a species into a previously occupied area, are commonly implemented where species of conservation concern have been extirpated. For example, Chiricahua leopard frogs (*Lithobates chiricahuensis*) were extirpated from the Altar Valley of Arizona, including the Buenos Aires National Wildlife Refuge in 2002, following a severe drought. To protect this Threatened species (U.S. Fish and Wildlife Service 2002, U. S. Fish and Wildlife Service 2011b), managers reintroduced captive-reared Chiricahua leopard frogs to the wildlife refuge in 2003 (Jarchow et al. 2016), with successful expansion in following years (Chandler et al. 2015).

Habitat alterations are a form of intervention that may benefit biodiversity more broadly than the species-specific efforts associated with supplementation and reintroductions. Alterations that create novel habitats that differ from historical conditions may be considered a *directing* action (e.g., Fig. 2). Yet to date, habitat alterations in protected areas have largely focused on *resisting* loss of biodiversity by restoring ecological and hydrologic processes to a historical state. Dam removal projects are perhaps the most high-profile example and have occurred in wilderness, national parks, and immediately downstream of wild and scenic river designations to restore fish passage, habitat connectivity, hydrologic function, and the homelands and cultural identities of local Indigenous peoples (The Elwha Act P.L.102-495, National Park Service 2009, National Oceanic and Atmospheric Administration 2024, U.S. Forest Service 2024). For example, the Glines Canyon and Elwha Dams, located within and downstream of Olympic National Park in Washington, inundated homelands of the Elwha Klallam Tribe along the Elwha River and extirpated culturally and economically relevant fish species from the national park (Duda et al. 2008, Pess et al. 2008). Removal of both dams between 2011–2014 restored hydrologic function of the river and allowed recolonization of more than a half dozen fish species, including upstream into portions of the Daniel J. Evan Wilderness within Olympic National Park (Duda et al. 2021). Other examples of habitat alteration in protected areas include interventions to address negative effects of historic and ongoing land use (e.g., Sullivan et al. 2014, Cowdery et al. 2019), resource extraction (e.g., Madej et al. 2006, Henderson et al. 2018), loss of beavers and beaver dams (Bartush 2021), and acid rain (see Box 2).

Removal of introduced species is an increasingly common intervention in protected areas to protect native fish species or to restore a historically fishless state (e.g., Liss et al. 2002, Vredenburg 2004, Syslo et al. 2011, Schnee et al. 2021). Restoring systems to a fishless state reduces recreational fishing opportunities; however, studies show increasing societal support for the benefits to amphibian and invertebrate taxa (Chiapella et al. 2018). It is important to note that not all removal efforts are focused on eradicating species that were intentionally introduced- volitional expansion of invasive species may also warrant interventions. Some examples specific to aquatic systems include removal of bull frogs (*Lithobates catesbeianus*) in Yosemite National Park and Wilderness in California (Kamoroff et al. 2020), and Burmese pythons (*Python bivittatus*) and Cuban treefrogs (*Osteopilus septentrionalis*) in Everglades National Park in Florida, including the Marjory Stoneman Douglas Wilderness Area (Rice et al. 2011, Leatherman 2022).

*Resisting* interventions are often combined to remove or address threats and restore biodiversity. In Great Smoky Mountain National Park along the Tennessee-North Carolina border, eradication of introduced rainbow trout was followed by reintroductions of native brook trout to speed population expansion and recovery in headwater streams (Kanno et al. 2016). In other cases, *resisting* interventions may require a balance of seemingly opposing mandates for management of the protected area (e.g., “natural” versus “untrammeled” and “undeveloped” characters of wilderness) in a context specific manner (Kelly and Landres 2023). For example, the use of preexisting human-made stock ponds (a human development/installation in wilderness) was critical to the success of Chiricahua leopard frogs in the Buenos Aires National Wildlife Refuge (Chandler et al. 2015).

In summary, common *resisting* actions to preserve biodiversity in protected areas may be categorized as those that conserve imperiled native species, restore habitat to a previous condition, or remove nonnative species. The methods for achieving these objectives are diverse because they are tailored to the history and circumstances of the freshwater system in a particular protected area. As climate change pushes freshwater systems further from historical baselines, managers may increasingly find themselves in situations where interventions to *resist* loss of freshwater biodiversity become too costly or ineffective. Under these circumstances managers may be forced to manage protected areas with freshwater systems with characteristics that differ from their historical baselines.

Box 2 *Resist*- Watershed liming to mitigate stream acidification in the St. Mary’s Wilderness Burning of fossil fuels, particularly coal, throughout the 19^th^ and 20^th^ centuries released large amounts of sulfuric and nitric acid into the atmosphere, ultimately causing acid rain and acidification of freshwater ecosystems (Driscoll and Wang 2019). This phenomenon has been particularly problematic in the eastern half of the United States where it has contributed to broadscale declines in freshwater biodiversity (Lovett et al. 2009, Driscoll and Wang 2019), including the regionally iconic eastern brook trout (*Salvelinus fontinalis*) (Hudy et al. 2008). Reduced reliance on fossil fuels (primarily coal) in the later 20th century has reduced acid deposition, allowing aquatic pH levels in some regions of North America and Europe to recover (Stoddard et al. 2003, Driscoll and Wang 2019). However, certain regions, including the southeastern United States, have experienced delayed recovery due to various factors such as water and soil chemistry (Stoddard et al. 2003, Driscoll and Wang 2019).

The St. Mary’s Wilderness located on the George Washington and Jefferson National Forests in Virginia has been particularly affected by stream acidification. By 1998, pH levels in streams and rivers of the wilderness had declined from a historic baseline of 6.8 to a range of 4.9–5.6, resulting in extirpation of many aquatic species (Mohn et al. 2004). To neutralize acidic compounds and raise pH to levels more suitable for native biota, managers of the St. Mary’s Wilderness applied calcium carbonate (in the form of limestone sand) to over 16 river km of the upper St. Mary’s River watershed in 1999 (Lieberman et al. 2013). Monitoring showed increases in species richness, population densities, and distributions of fish and macroinvertebrates in the St. Mary’s watershed following treatment (Mohn et al. 2004). These benefits included increased densities of native brook trout, which supported opportunities for recreational fishing in the wilderness area. Yet, as expected, the pH level and biodiversity began to decline after several years as the limestone was “consumed” in the system (Norman et al. 2005, Williams and Downey 2010). To maintain pH levels, managers repeated liming efforts in the St. Mary’s Wilderness in 2005, 2013, and 2022. Monitoring following all liming efforts demonstrated a consistent positive response of aquatic biodiversity for five to seven years after each treatment, followed by declines as limestone was consumed. In the record of decision for the 2013 treatment, the Regional Forester noted the necessity for repeated intervention to protect freshwater biodiversity and wilderness character (U.S. Forest Service 2011):

*I also acknowledge that at some time in the future, additional liming may be needed to the same streams… Liming does not solve the larger problem. It serves to keep aquatic species alive until air pollution is decreased. The question is whether to allow continued loss of the aquatic biota while preserving the wilderness concept or ideal of “untrammeled”, or compromise the wilderness ideal, to preserve the aquatic resource? I maintain that society has already compromised eastern wilderness values by allowing air polluting emissions to continue that lead to acid precipitation. In my judgment, liming will not further compromise the wilderness values of St. Mary’s, but instead will help to preserve one of the values that led to its wilderness designation in the first place*.

As the reservoir of acidic compounds is gradually depleted, acid deposition has slowed in the St. Mary’s River basin and repeated liming may no longer be necessary to maintain suitable pH levels following the 2022 treatment (Good et al. 2023).

### Directing Freshwater Biodiversity in Protected Areas

Management interventions that *direct* differ from those that *resist* in that the outcomes support an ecological state that differs from current or historical baselines (Fig. 2). Because protected areas were designated with historical states in mind (e.g., maintaining “primeval character” of federally designated wilderness areas), *directing* actions may be perceived as inappropriate (Heller and Zavaleta 2009, Cravens et al. 2024), especially if there is no precedent for the ecological baseline elsewhere. As a result, *directing* actions have rarely been implemented in protected areas. Yet, as climate driven transformation makes restoring freshwater systems to historical baselines more difficult (e.g., Poff 2018), *directing* actions may become more common.

The most common *directing* actions taken to conserve freshwater biodiversity in protected areas focus on translocations of regionally native species into local habitats where they were not historically present. For example, California red-legged frogs (*Rana draytonii*) have been extirpated from over 70% of their historic range, leading to listing as Threatened under the Endangered Species Act (U.S. Fish and Wildlife Service 1996). To protect the species and create a “safe harbor” population for future conservation efforts, managers introduced red-legged frogs to Yosemite National Park, including portions designated as wilderness (Adams et al. 2023b), despite uncertainty of the species’ historical presence in the park (Adams et al. 2023a). Managers noted that the decision to introduce red-legged frogs potentially outside their historical range within Yosemite National Park combined all available historical baseline information with “contemporary realities” to facilitate climate adaptation and biodiversity conservation throughout the Yosemite Valley (Adams et al. 2023a). Similar translocations have occurred in other national parks, such as translocations of Threatened bull trout (*Salvelinus confluentus*) (U. S. Fish and Wildlife Service 1999) from lower elevations of Glacier National Park to climate refugia above waterfalls where they were not historically present.

The *directing* interventions above represent a tradeoff among ecological values because they favor preservation of a focal species while altering the recipient ecosystem. *Directing* actions may also be guided by tradeoffs among broader values, such as a need to balance socio-cultural and economic values with management of freshwater systems (Box 3). Altering systems away from ecological baselines is risky as it may result in unintended outcomes. Studies show that unintended outcomes have primarily occurred when management actions favor socio-cultural and economic values without conservation principles in mind (Novak et al. 2021; see also the discussion on fish stocking in Section II). Yet even when management actions are centered on ecological values, the effects of climate driven transformation introduce greater uncertainty in the ability of protected areas to support biodiversity (Hoffmann 2022). Combining the RAD framework can provide a more holistic structure to help balance diverse and competing values with uncertainty in management outcomes when responding to freshwater biodiversity loss (Sesser et al. 2025).

Box 3 *Direct*- Stocking a regionally native fish in historically fishless lakes following invasive species eradication Recreational angling is a valued pastime in Montana, supporting a nearly US$1 billion annual economy for the state (Headwater Economics 2018). Westslope cutthroat trout (WCT, *Oncorhynchus lewisi*) are the state fish of Montana and a popular game species throughout the region. Once prevalent throughout the northern Rocky Mountains, WCT have suffered range-wide declines from interactions with nonnative species (especially hybridization with congeneric species) and human activities that degrade habitat and isolate populations (Shepard et al. 2005). As a result, they are considered a species of conservation concern in every U.S. state throughout their native range and are listed under the Species at Risk Act in Canada (S.C. 2002, c. 29).

The South Fork Flathead River flows through the Flathead National Forest of western Montana. A majority of the river basin is located within the Bob Marshall Wilderness Area, and nearly 70 km of the mainstem is designated as a wild and scenic river (https://www.rivers.gov/rivers/river/flathead). The basin supports more than 50% of the nonhybridized and interconnected populations of WCT in Montana (Shepard et al. 2005) and is projected to provide suitable habitat for WCT under future climate scenarios (Isaak et al. 2015). As a result, WCT of the South Fork Flathead River basin are considered a regional stronghold (Likens 1984, Grisak and Marotz 2002). Throughout the early to mid-20th century, nonnative rainbow and Yellowstone cutthroat trout (*O. mykiss* and *O. clarkii bouvieri*, respectively) were stocked throughout the South Fork Flathead River basin legally (in early undocumented efforts by fisheries managers prior to 1940) and illegally (by members of the public) to support recreational angling opportunities (Grisak and Marotz 2002). As management shifted towards a focus on native species in the 1980s, Montana Fish, Wildlife and Parks conducted a thorough review of WCT status in the South Fork River basin. Assessments identified hybridization with nonnative trout as the primary threat to the WCT stronghold (Sage 1993), and that risk was highest downstream of historically fishless lakes that had been stocked with nonnative trout (Likens 1984, Sage 1993). In the early 2000s, Montana Fish, Wildlife and Parks and the U.S. Forest Service developed and implemented an interagency conservation plan to preserve nonhybridized WCT in the South Fork Flathead River basin (Bonnville Power Administration 2006). This plan focused on chemical treatments to eradicate nonnative trout from 21 historically fishless lakes, including eight in the Bob Marshall Wilderness Area (Bonneville Power Administration 2006).

Public comments collected on the conservation plan emphasized concern for the loss of recreational angling opportunities and associated impacts to commercial outfitters and local tourism caused by nonnative trout removals from focal lakes (Bonneville Power Administration 2005). Considering the socio-cultural and economic value of the fishery, managers chose to restock these lakes with WCT collected from donor populations within the Bob Marshall Wilderness Area (Montana Fish Wildlife and Parks 2004). The conservation plan was implemented over a ten-year period beginning in 2007 (Grisak and Marotz 2002). Managers incorporated recreational and economic values into the scheduling and sequencing of treatments and restocking to minimize disruption to angling opportunities and hardships to local outfitters and guides (M. Boyer, pers. comm.).

Had managers removed nonnative fish and restored these lakes to a fishless state, this would be considered a *resisting* intervention. There are no species of conservation concern that rely on fishless lakes in the basin for survival, and the Bob Marshall Wilderness Area includes other lakes that remain in a historically fishless condition (Grisak and Marotz 2002). The decision to *direct* the freshwater community in these lakes away from a historical baseline represents a careful balance between promoting social-cultural and economic values of recreational angling with minimizing risks to native WCT in the South Fork Flathead River basin. Furthermore, by sourcing local WCT for stocking, the populations established in these lakes serve as a genetic reserve to support future conservation efforts in western Montana.

## Section IV: Considerations for the RAD Framework and the Future of Freshwater Biodiversity

The decision to *resist*, *accept*, or *direct* ecological transformation is not a new concept in natural resources management. Yet, examples of applying the RAD framework to freshwater ecosystems or protected areas are limited. Our retrospective examination demonstrates how freshwater biodiversity managers are already familiar with RAD concepts and how these concepts may be used to balance conflicting mandates in protected areas. When applied in a forward-looking manner, the RAD framework can help identify management pivot points and outline proactive approaches to respond to increasingly uncertain and dynamic ecological conditions (Lynch et al. 2025).

Choosing among competing management approaches requires consideration of context specific socio-cultural, economic, and ecological values. Moreover, the feasibility of each approach within protected areas may be influenced by legal mandates (Cravens et al. 2024). For example, the Wilderness Act of 1964 requires managers to minimize human influence but also notes that human interventions may be performed if necessary to preserve overall wilderness character (16 U.S.C. 1133 (c)). *Resisting* or *directing* interventions to protect freshwater biodiversity may receive greater support in a wilderness context if they are linked to other federal legislation, such as the Endangered Species Act of 1973. Several laws also mandate consideration of socio-cultural values of rightsholders. For instance, federal agencies are required to consult with Native American Tribes prior to taking any action that would directly affect Tribal treaties and cultural or religious resources (see Advisory Council on Historic Preservation 2021, Congressional Research Service 2024). In these contexts, the RAD framework provides a shared vocabulary to foster collaboration and communication among managers, rightsholders, and stakeholders (Schuurman et al. 2022). This is particularly important for freshwater species and ecosystems where management decisions have both immediate and long-term consequences for partners with differing priorities and cultural values (e.g., Shultz et al. 2022). By encouraging creative thinking around ecological transformation, the RAD framework can help managers and their partners develop a broader, more inclusive decision space.

The RAD framework is designed to be flexible to facilitate creative responses to ecological transformation. For example, managers may choose one or more approaches that do not precisely fit within a single *resist*, *accept*, or *direct* category, or apply multiple approaches across different parts of a protected area (e.g., Ward et al. 2023). Additionally, collaborative management across protected area boundaries may promote preservation of freshwater biodiversity at ecologically relevant scales, while balancing conflicting mandates of different protected area types. For example, strategically placed interventions that *resist* or *direct* biodiversity loss outside of federally designated wilderness (e.g., dam removal, introductions of imperiled species, etc.) can provide spillover benefits that preserve “natural” character while avoiding human manipulation within the protected area boundary. Furthermore, expanding collaborations with stewards of non-federal lands (e.g., Tribes, local governments, land trusts, and private landowners) can enhance benefits of the protected area network, especially in the western United States where ecological connectivity across landscapes remains relatively high (Belote et al. 2016).

Moving beyond localized and single-species management towards collaborative, landscape-level efforts will benefit freshwater biodiversity and processes more holistically (Saunders et al. 2002, Finlayson et al. 2018, Acreman et al. 2020). Yet, localized intervention will still be necessary to mitigate biodiversity losses, particularly in freshwater systems facing current, identifiable threats (Cowx and Aya 2011, Acreman et al. 2020, Dudgeon and Strayer 2025). In this context, we caution that our current management challenges are often the consequence of past intervention successes (see Section III). The RAD framework offers a structured way to consider a diverse range of values, clarify tradeoffs, and adopt proactive strategies that improve decision-making for freshwater biodiversity in the context of ongoing change. Through this broader decision space, we may be able to select management approaches that support freshwater biodiversity and serve the values of both current and future generations.

## Supplementary information

TableS1

TableS2

## Data Availability

No datasets were generated or analysed during the current study.

## References

[CR1] Acreman M, Hughes KA, Arthington AH, Tickner D, Dueñas MA (2020) Protected areas and freshwater biodiversity: a novel systematic review distils eight lessons for effective conservation. Conserv Lett 13: e12684

[CR2] Adams AJ, Brown KC, Jennings MR, Grasso RL (2023a) Homecoming or new pad: Historical evidence for California red-legged frogs and other amphibians in the Yosemite Region, California. Northwest Naturalist 104:1–25

[CR3] Adams AJ, Grasso RL, Mazur RL (2023b) Safe harbor: translocating California red-legged frogs to a climate refuge in Yosemite National Park. Anim Conserv 26:606–608. 10.1111/acv.12863

[CR4] Advisory Council on Historic Preservation, (2021) Consultation with Indian Tribes in the Section 106 review process: The handbook. Washington, D.C. https://www.achp.gov/sites/default/files/2021-06/ConsultationwithIndianTribesHandbook6-11-21Final.pdf. Accessed 17 December 2024

[CR5] Aplet GH, Mckinley PS (2017) A portfolio approach to managing ecological risks of global change. Ecosyst Health Sustainability 3: e01261

[CR6] Arlinghaus R et al. (2021) Global participation in and public attitudes toward recreational fishing: International perspectives and developments. Rev Fish Sci Aquac 29(1):58–95. 10.1080/23308249.2020.1782340

[CR7] Asadieh B, Krakauer NY (2017) Global change in streamflow extremes under cliamte change over the 21^st^ century. Hydrol Earth Syst Sci 21:5863–5874

[CR8] Association of Fish and Wildlife Agencies, (2006) Policies and guidelines for fish and wildlfe managemnet in National Forests and Bureau of Land Management wilderness. p 17. https://www.blm.gov/policy/im-2007-052. Accessed 17 December 2024

[CR9] Bartush NC, (2021) Using beaver dam analogs to restore riparian ecosystems influenced by large ungulates: a review for the southern Rocky Mountains. Master of Science, Colorado State University. https://hdl.handle.net/10217/233604

[CR10] Bash JS, Ryan CM (2002) Stream restoration and enhancement projects: is anyone monitoring?. Environ Manag 29:877–885 10.1007/s00267-001-0066-3 11992178

[CR11] Belote RT et al. (2016) Identifying corridors among large protected areas in the United States. PLOS One 11:e0154223 27104683 10.1371/journal.pone.0154223 PMC4841590

[CR12] Bonneville Power Administration, (2005) South Fork Flathead watershed westslope cutthroat trout conservaiton program final environmental impact statement. Portland, OR. https://www.energy.gov/sites/default/files/nepapub/nepa_documents/RedDont/EIS-0353-FEIS-2005.pdf

[CR13] Bonneville Power Administration, (2006) South Fork Flathead watershed westslope cutthroat trout conservation program, Record of decision. Portland, OR. https://www.bpa.gov/-/media/Aep/about/publications/records-of-decision/2006-rod/rod-20060501-south-fork-flathead-watershed-westslope-cutthroat-trout-conservation-program.pdf

[CR14] Bower SD, Lennox RJ, Cooke SJ (2015) Is there a role for freshwater protected areas in the conservation of migratory fish?. Inland Waters 5:1–6

[CR15] Brodie JF et al. (2023) Landscape-scale benefits of protected areas for tropical biodiversity. Nature 620:807–812 37612395 10.1038/s41586-023-06410-z

[CR16] Brown K (2021) An exceedingly simple, little ecosystem: Devil’s Hole, endangered species conservaiton and scientific environments. Notes Rec 75:239–257

[CR17] Brown TC, Froemke P, Mahat V, Ramirez JA, (2016) Mean annual renewable water supply of the contiguous United States. U.S. Department of Agriculture, Forest Service, Rocky Mountain Research Station, Fort Collins. https://research.fs.usda.gov/sites/default/files/2023-04/rmrs-mean_annual_renewable_water_supply_of_the_contiguous_united_states_briefing_paper.pdf

[CR18] Chandler RB, Muths E, Sigafus BH, Schwalbe CR, Jarchow CJ, Hossack BR (2015) Spatial occupancy models for predicting metapopulation dynamics and viability following reintroduction. J Appl Ecol 52:1325–1333

[CR19] Chiapella AM, Nielsen-Pincus M, Strecker AL (2018) Public perceptions of mountain lake fisheries management in national parks. J Environ Manag 226:169–179 10.1016/j.jenvman.2018.08.040 30119041

[CR20] Clifford KR, Cravens AE, Knapp CN (2021) Responding to ecological transformation: Mental models, external constraints, and manager decision-making. BioScience 72:57–70

[CR21] Congressional Research Service, (2024) Federal-Tribal consultation: Background and issues for Congress. https://crsreports.congress.gov/product/pdf/R/R48093 Accessed 17 December 2024

[CR22] Cole DN, Landres PB (1996) Threats to Wilderness Ecosystems: Impacts and Research Needs. Ecol Appl 6:168–184

[CR23] Cole DN, Yung L, (2012) Beyond naturalness: rethinking park and wilderness stewardship in an era of rapid change. Island Press, Washington D.C.

[CR24] Colvin SM et al. (2019) Headwater streams and wetlands are critical for sustaining fish, fishereis, and ecosystem services. Fisheries 44:73–91

[CR25] Cowdery TK, Christenson CA, Ziegeweid JR, (2019) The hydrologic benefits of wetland and prairie restoration in western Minnesota—lessons learned at the Glacial Ridge National Wildlife Refuge, 2002–15 US Geological Survey Scientific Investigations Report. U.S. Geological Survey, p 81. https://pubs.usgs.gov/sir/2019/5041/sir20195041.pdf. Accessed 17 December 2024

[CR26] Cowx IG, Aya MP (2011) Paradigm shifts in fish conservation: moving to the ecosystem services concept. J Fish Biol 79:1663–1680 22136245 10.1111/j.1095-8649.2011.03144.x

[CR27] Cravens AE, Clifford KR, Knapp C, Travis WR, (2024) The dynamic feasibility of resisting (R), accepting (A), or directing (D) ecological change. Conservation Biology e14331. 10.1111/cobi.14331 10.1111/cobi.14331 PMC11959322 39016709

[CR28] Cucherousset J, Olden JD (2011) Ecological impacts of nonnative freshwater fishes. Fisheries 36:215–230

[CR29] Detmer TM et al. (2025) Community-wide transient dynamics of lake fish populations in response to two decades of supressing an introduced predator. J Appl Ecol 62:1406–1420

[CR30] Dinerstein E et al. (2019) A Global Deal For Nature: Guiding principles, milestones, and targets. Sci Adv 5: eaaw2869 31016243 10.1126/sciadv.aaw2869 PMC6474764

[CR31] Driscoll CT, Wang Z, (2019) Ecosystem effects of acidic deposition. In: Maurice P (ed) Encyclopedia of Water: Science, Technology, and Society. Wiley Online Library. 10.1002/9781119300762.wsts0043

[CR32] Duda JJ, Freilich JE, Schreiner EG (2008) Baseline studies in the Elwha River ecosystem prior to dam removal: introduction to the special issue. Northwest Sci 82:1–12

[CR33] Duda JJ et al. (2021) Reconnecting the Elwha River: spatial patterns of fish response to dam removal. Front Ecol Evol 9:765488

[CR34] Dudgeon D et al. (2006) Freshwater biodiversity: importance, threats, status and conservation challenges. Biol Rev 81:163–182 16336747 10.1017/S1464793105006950

[CR35] Dudgeon D, Strayer DL (2025) Bending the curve of global freshwater biodiversity loss: what are the prospects?. Biol Rev 100:205–226 39221642 10.1111/brv.13137 PMC11718631

[CR36] Dudley N, Stolton S, (2008) Defining protected areas: An international conference in Almeria, Spain. IUCN, Switzerland. https://portals.iucn.org/library/sites/library/files/documents/2008-106.pdf. Accessed 17 December 2024

[CR37] Dunham JB, Pilliod DS, Young MK (2004) Assessing the consequences of non-native trout in headwater ecosystems in Western North America. Fisheries 29:18–16

[CR38] Eby L, Roach WJ, Crowder LB, Standford JA (2006) Effects of stocking- up freshwater food webs. Trends Ecol Evol 21:576–584 16828522 10.1016/j.tree.2006.06.016

[CR39] Epifanio JM, Waples RS, (2016) Artificial propagation of freshwater fishes: Benefits and risks to recipient ecosystems from stocking, translocation, and re-introduction. In: Closs GP, Krkosek M, Olden JD (eds) Conservation of Freshwater Fishes. Cambridge University Press, Cambridge pp 339-436

[CR40] Ettling JA, Wanner MD, Pedigo AS, Kenkel JL, Noble KR, Briggler JT (2017) Augmentation programme for the endangered Ozark hellbender Cryptobranchus alleganiensis bishopi in Missouri. Int Zoo Yearb 51:79–86. 10.1111/izy.12162

[CR41] Ettling JA, Wanner MD, Schuette CD, Armstrong SL, Pedigo AS, Briggler JT(2013)Captive reproduction and husbandry of adult Ozark hellbenders,Cryptobranchus alleganiensis bishopi.Herpetol Rev44:605–610

[CR42] Finlayson CM, Arthington AH, Pittock J, (2018) Freshwater ecosystems in protected areas. Taylor and Francis, London. 1st Edition. 10.4324/9781315226385

[CR43] Galloway BT et al. (2016) A framework for assessing the feasibility of native fish conservation translocations: applications to threatened Bull Trout. North Am J Fish Manag 36:754–768.

[CR44] Gartner WC, Love LL, Erkkila D, Fulton DC, (2002) Economic impacts and social benefits of coldwater angling in Minnesota. University of Minnesota - Extension Service, St. Paul, MN. https://files.dnr.state.mn.us/fisheries/management/coldwateranglingreport.pdf. Accessed 17 December 2024

[CR45] Giam X, Olden JD, Simberloff D (2018) Impact of coal mining on stream biodiversity in the U.S. and its regulatory implications. Nat Sustainability 1:176–183

[CR46] Goetz SJ, Jantz P, Jantz CA (2009) Connectivity of core habitat in the Northeastern United States: Parks and protected areas in a landscape context. Remote Sens Environ 113:1421–1429

[CR47] Good SA, Williams NC, Downey DM, (2023) Water chemistry of the St. Mary’s River and tributaries: Assessment of acid rain mitigation (liming) 1999-2022. James Madison University, Harrisonberg, VA, p 26

[CR48] Gray CL et al. (2016) Local biodiversity is higher inside than outside terrestrial protected areas worldwide. Nat Commun 7(1):12306. 10.1038/ncomms12306 27465407 10.1038/ncomms12306 PMC4974472

[CR49] Grisak G, Marotz B, (2002) South Fork Flathead watershed westslope cutthroat trout conservation program. Project No. 1991-01903. BPA Report DOE/BP-00005043-1. https://www.nwcouncil.org/sites/default/files/App74_MountLakes_rpt.pdf. Accessed 17 December 2024

[CR50] Halverson A, (2010) An entirely synthetic fish: how rainbow trout beguiled America and overran the world. Yale University Press, New Haven

[CR51] Headwater Economics, (2018) Outdoor Recreation and Montana’s Economy Report. Montana Office of Outdoor Recration Helena, MT. https://headwaterseconomics.org/wp-content/uploads/montana-outdoor-recreation-economy-report.pdf. Accessed 17 December 2024

[CR52] Heller NE, Zavaleta ES (2009) Biodiversity management in the face of climate change: A review of 22 years of recommendations. Biol Conserv 142:14–32

[CR53] Henderson T et al. (2018) Mine tailings reclamation project improves water quality in Yellowstone’s Soda Butte Creek. Park Sci 34:1–20.

[CR54] Herbst DB, Bogan MT, Roll SK, Safford HD (2012) Effects of livestock exclusion on in-stream habitat and benthic invertebrate assemblages in montane streams. Freshw Biol 57:204–217

[CR55] Hogg JT, Forbes SH, Steele BM, Luikart G (2006) Genetic rescue of an insular population of large mammals. Proc R Soc B: Biol Sci 273:1491–1499 10.1098/rspb.2006.3477 PMC1560318 16777743

[CR56] Holmes TP, (2022) Accounting for wilderness economic values in a historical, cultural, and social context. In: Holmes TP (ed) A perpetual flow of benefits: wilderness economic values in an evolving, multicultural society. WO-GTR-XXX. U.S. Department of Agriculture, Forest Service, Washington Office, Washington D.C. pp 1-24. 10.2737/WO-GTR-101

[CR57] Holmlund CM, Hammer M (2004) Effects of fish stocking on ecosystem services: An overview and case study using the Stockholm Archipelago. Environ Manag 33:799–820 10.1007/s00267-004-0051-8 15156349

[CR58] Hoffmann S (2022) Challenges and opportunities of area-based conservation in reaching biodiversity and sustainability goals. Biodivers Conserv 31:325–352

[CR59] Hossack BR et al. (2015) Trends in Rocky Mountain amphibians and the role of beaver as a keystone species. Biol Conserv 187:260–269

[CR60] Hubbard KD, Nixon M, Smith JA (2000) The Wilderness Act’s Impact on Mining Activities: Policy Versus Practice. Public Lands Resour Law Dig 37:11

[CR61] Hudy M, Thieling TM, Gillespie N, Smith EP (2008) Distribution, status, and land use characteristics of subwatersheds within the native range of brook trout in the eastern United States. North Am J Fish Manag 28:1069–1085

[CR62] Isaak DJ, Young MK (2023) Cold-water habitats, climate refugia, and their utility for conserving salmonid fishes. Can J Fish Aquat Sci 80:1187–1206

[CR63] Isaak DJ, Young MK, Nagel DE, Horan DL, Groce MC (2015) The cold-water climate shield: delineating refugia for preserving salmonid fishes through the 21st century. Glob Change Biol 21(7):2540–2553 10.1111/gcb.12879 25728937

[CR64] Jacobson PC, Stefan HG, Pereira DL (2010) Coldwater fish oxythermal habitat in Minnesota lakes: influence of total phosphorus, July air temperature, and relative depth. Can J Fish Aquat Sci 67:2002–2013

[CR65] Jarchow CJ, Hossack BR, Sigafus BH, Schwalbe CR, Muths E (2016) Modeling Habitat Connectivity to Inform Reintroductions: A Case Study with the Chiricahua Leopard Frog. J Herpetol 50:63–69

[CR66] Johnson WP, Beauchamps JS, Butler MJ, Sanchez JI (2022) Successful establishement of endangered springsnails following habitat enhancement and translcoations. Aquat Conservaiton: Mar Freshw Ecosyst 32:1249–1262

[CR67] Jones EBD, Helfman GS, Harper JO, Bolstad PV (1999) Effects of riparian forest removal on fish assemblages in southern Appalachian streams. Conserv Biol 13:1454–1465

[CR68] Jones R et al. (2013) Climate change impacts on freshwater recreational fishing in the United States. Mitig Adapt Strateg Glob Change 18:731–758

[CR69] Kamoroff C, Daniele N, Grasso RL, Rising R, Espinoza T, Goldberg CS (2020) Effective removal of the American bullfrog (*Lithobates catesbeianus*) on a landscape level: long term monitoring and removal efforts in Yosemite Valley, Yosemite National Park. Biol Invasions 22(2):617–626

[CR70] Kanno Y, Kulp MA, Moore SE (2016) Recovery of native brook trout populations following the eradication of nonnative rainbow trout in southern Appalachian Mountains streams. North Am J Fish Manag 36:1325–1335

[CR71] Kelly P, Landres P (2023) Does wilderness matter in the Anthropocene? Resolving a fundamental dilemma about the role of wilderness in 21st century conservation. Ethics, Policy Environ 26:422–437

[CR72] Kershner JL, Roper BB, Bouwes N, Henderson R, Archer E (2004) An analysis of stream habitat conditions in reference and managed watersheds on some federal lands within the Columbia River basin. North Am J Fish Manag 24:1363–1375

[CR73] LaFrance BJ, Ray AM, Tercek MT, Fisher RN, Hossack BR (2024) Amphibian richness, rarity, threats, and conservation prospects across the U.S. National Park System. npj Biodivers 3:35 39572683 10.1038/s44185-024-00067-1 PMC11582706

[CR74] Landres P, et al. (2015) Keeping it wild 2: An updated interagency strategy to monitor trends in wilderness character across the National Wilderness Preservation System. RMRS-GTR-340. U.S. Department of Agriculture, Forest Service, Rocky Mountain Research Station, Fort Collins. 10.2737/RMRS-GTR-340

[CR75] Landres P, Meyer S, Matthews S (2001) The Wilderness Act and fish stocking: An overview of legislation, judicial interpretation, and agency implementation. Ecosystems 4:287–295

[CR76] Landres PB, Marsh S, Merigliano L, Ritter D, Norman A, (1998) Boundary effects on wilderness and other natural areas. In: Kinght RL, Landres PB (eds) Stewardship Across Boundaries. Island Press, Washington, D.C. p 117-139

[CR77] Lawrence DJ, Larson ER, Liermann CAR, Mims MC, Pool TK, Olden JD (2011) National parks as protected areas for U.S. freshwater fish diversity. Conserv Lett 4:364–371

[CR78] Leatherman SP (2022) Management of invasive snakes in coastal environments: A baseline assessment of the Burmese python invasion in the Florida Everglades. Mar Pollut Bull 182: 113996 35921734 10.1016/j.marpolbul.2022.113996

[CR79] Lieberman L, Hahn B, Carlson A, (2013) Saint Mary’s Wilderness: Depositing limestone sand in the Saint Mary’s River to buffer the effects of acid deposition on aquatic organisms. U.S. Forest Service, Missoula, MT. https://winapps.umt.edu/winapps/media2/wilderness/toolboxes/documents/restoration/FS_Saint%20Mary’s%20Wilderness%20Case%20Study.pdf. Accessed 17 December 2024

[CR80] Lieberman K, Hahn B, Landres P (2018) Manipulating the wild: a survey of restoration and management interventions in U.S. wilderness. Restor Ecol 26:900–908

[CR81] Likens GA (1984) The present status and distribution of the westslope cutthroat trout (*Salmo clarki lewisi*) east and west of the Continental Divide in Montana. Montana Fish, Wildlife, and Parks, Helena, MT.

[CR82] Liss WJ, Larson GL, Hoffman RL, (2002) Ecological impacts of introduced trout on native aquatic communities in mountain lakes. Phase III Final Report. North Cascades Natioanl Park Complex. U.S. Geological Survey, Forest and Rangeland Ecosystem Science Center, Corvallis, OR. https://www.govinfo.gov/app/details/GOVPUB-I29-PURL-gpo16781. Accessed 17 December 2024

[CR83] Loomis E (2017) Forests and logging in the United States. Oxford Research Encyclopedia of American History. 10.1093/acrefore/9780199329175.013.188

[CR84] Lovett GM et al. (2009) Effects of air pollution on ecosystems and biological diversity in the eastern United States. Ann N Y Acad Sci 1162:99–135 19432647 10.1111/j.1749-6632.2009.04153.x

[CR85] Lynch AJ et al. (2021a) Managing for RADical ecosystem change: applying the Resist-Accept-Direct (RAD) framework. Front Ecol Environ 19:461–469

[CR86] Lynch AJ et al. (2021b) RAD Adaptive Management for Transforming Ecosystems. BioScience 72:45–56

[CR87] Lynch AJ et al. (2025) RAD (resist-accept-direct) switch points and triggers for adaptation planning. J Environ Manag 392: 126419 10.1016/j.jenvman.2025.126419 40774859

[CR88] Madej MA, Ambrose H, Currens C, Hadden S (2006) Assessing changes in stream health following watershed restoration: A 30-year perspective, Redwood Creek Basin, Humboldt County, California. U.S. Geological Survey, Arcata, CA. 10.13140/RG.2.1.1692.3286

[CR89] Magness DR et al. (2022) Management Foundations for Navigating Ecological Transformation by Resisting, Accepting, or Directing Social–Ecological Change. Bioscience 72:30–44

[CR90] Matthews OP, Haak A, Toffenetti K (1985) Mining and wilderness: Incompatible uses or justifiable compromise? Environ: Sci Policy Sustain Dev 27:12–36

[CR91] Meretsky VJ et al. (2006) New directions in conservation for the National Wildlife Refuge System. BioScience 56:135–143

[CR92] Milly PC et al. (2008) Stationarity is dead: Whither water management?. Science 319:573–574 18239110 10.1126/science.1151915

[CR93] Minnesota Department of Natural Resources (1998) Lake survey report: East Bearskin Lake. https://www.dnr.state.mn.us/lakefind/showreport.html?downum=16014600. Accessed 8 January 2025

[CR94] Minnesota Department of Natural Resources (2023) Lake survey report: East Bearskin Lake. https://www.dnr.state.mn.us/lakefind/showreport.html?downum=16014600. Accessed 8 January 2025

[CR95] Minnesota Department of Natural Resources and U.S. Forest Service (2020) Memorandum of understanding for fisheries management within the Boundary Waters Canoe Area Wilderness.

[CR96] Miqueleiz I, Ariño AH, Miranda R (2023) Spatial priorities for freshwater fish conservation in reltation to protected areas. Aquat Conserv: Mar Freshw Ecosyst 33:1028–1038

[CR97] Mohn LO, Bugas PE, Kirk DM, Downey DM (2004) Long-term results of mitigating stream acidification using limestone sand in St. Mary’s River, Virginia. In: Wild Trout Symposium VIII. Yellowstone National Park pp 214-221. https://www.wildtroutsymposium.com/proceedings-8.pdf

[CR98] Montana Fish Wildlife and Parks (2004) Hatchery and genetic management plan resident fish edition - Wild genetically pure westslope cutthroat trout. https://www.nwcouncil.org/sites/default/files/App80_HGMP_Sekokini_.pdf. Accessed 17 Decemebr 2024

[CR99] Moss WE et al. (2025) Applying the resist–accept–direct (RAD) framework to wildlife health management. BioScience biaf061 10.1093/biosci/biaf061

[CR100] National Oceanic and Atmospheric Administration (2024) World’s biggest dam removal project to open 420 miles of salmon habitat this fall. https://www.fisheries.noaa.gov/feature-story/worlds-biggest-dam-removal-project-open-420-miles-salmon-habitat-fall#:~:text=About%20Us-,World’s%20Biggest%20Dam%20Removal%20Project%20to%20Open,of%20Salmon%20Habitat%20this%20Fall&text=With%20the%20dams%20on%20the,funding%20for%20additional%20river%20restoratio. Accessed 17 December 2024

[CR101] National Park Service (2009) Point Reyes National Seashore completes restoration within the Phillip Burton Wilderness. https://www.nps.gov/pore/learn/nature/upload/planning_glenbrook_restoration_insidenps_091009.pdf

[CR102] National Park Service (2017) National Park Service system plan: One hundred years. https://www.nps.gov/subjects/parkplanning/upload/National-Park-System-Plan.pdf

[CR103] National Park Service (2018) Record of decision: Final enviornmental impact statement to address the presence of wolves at Isle Royale National Park. https://parkplanning.nps.gov/document.cfm?parkID=140&projectID=59316&documentID=88676

[CR104] Norman C, Elliot RC, Downey DM (2005) St. Mary’s Stream Water Chemistry (1999-2004) James Madison University, Harrisonberg, VA. https://www.fs.usda.gov/Internet/FSE_DOCUMENTS/fsbdev3_000164.pdf. Accessed 17 December 2024

[CR105] Novak BJ, Phelan R, Weber M (2021) U.S. cosnervation translocations: over a century of intended consquences. Conserv Sci Pr 21: e394

[CR106] O’Connor JE, Duda JJ, Grant GE (2015) 1000 dams and counting. Science 348:496–497 25931537 10.1126/science.aaa9204

[CR107] Panlasigui S, Davis AJS, Mangiante MJ, Darling JA (2018) Assessing threats of non-native species to native freshwater biodiversity: Conservation priorities for the United States. Biol Conserv 224:199–208 30245526 10.1016/j.biocon.2018.05.019 PMC6145479

[CR108] Parks SA, Holsinger LM, Abatzoglou JT, Littlefield CE, Zeller KA (2023) Protected areas not likely to serve as steppingstones for species undergoing climate-induced range shifts. Glob Change Biol 29:2681–2696 10.1111/gcb.16629 36880282

[CR109] Pess GR, McHenry ML, Beechie TJ, Davies J (2008) Biological impacts of the Elwha River dams and potential salmonid responses to dam removal. Northwest Sci 82:72–90

[CR110] Pister EP (2001) Wilderness Fish Stocking: History and Perspective. Ecosystems 4:279–286

[CR111] Poff NL (2018) Beyond the natural flow regime? Broadening the hydro-ecological foundation to meet environmental flows challenges in a non-stationary world. Freshw Biol 63:1011–1021

[CR112] Poff NL et al. (1997) The natural flow regime. BioScience 47:769–784

[CR113] Rahel FJ (2016) Changing philosophies of fisheries management as illustrated by the history of fishing regulations in Wyoming. Fisheries 41:38–48

[CR114] Rahel FJ, Lynch AJ (2022) Managing fisheries within a RAD framework: Concepts and applications. Fish Manag Ecol 29:323–328

[CR115] Reid AJ et al. (2019) Emerging threats and persisent conservation challenges for freshwater biodiversity. Biol Rev 94:849–873 30467930 10.1111/brv.12480

[CR116] Rice KG, Waddle JH, Miller MW, Crockett ME, Mazzotti FJ, Percival HF (2011) Recovery of native treefrogs after removal of nonindigenous Cuban treefrogs, *Osteopilus septentrionalis*. Herpetologica 67:105–117

[CR117] Roni P, Beechie T, Pess G, Hanson K (2015) Wood placement in river restoration: fact, fiction, and future direction. Can J Fish Aquat Sci 72:466–478

[CR118] Roper BB, Capurso JM, Paroz Y, Young MK (2018) Conservation of aquatic biodiversity in the context of multiple-use management on National Forest System lands. Fisheries 43:396–405. 10.1002/fsh.10168

[CR119] Rothlisberger JD, Scalley TH, Thurow RF (2017) The role of wild and scenic rivers in the conservation of aquatic biodiversity. Int J Wilderness 23:49–63

[CR120] Sage GK (1993) Allozymic and parasitic examination of interspecifific introgression in Oncorhynchus from the South Fork of the Flathead River drainage. Master of Arts, Univeristy of Montana. https://scholarworks.umt.edu/etd/6987

[CR121] Saunders DL, Meeuwig JJ, Vincent ACJ (2002) Freshwater Protected Areas: Strategies for Conservation. Conserv Biol 16:30–41. 10.1046/j.1523-1739.2002.99562.x 35701954 10.1046/j.1523-1739.2002.99562.x

[CR122] Scamardo JE, Marshall S, Wohl E (2022) Estimating widespread beaver dam loss: Habitat decline and surface storage loss at a regional scale. Ecosphere 13: e3962. 10.1002/ecs2.3962

[CR123] Schnee ME, Clancy NG, Boyer MC, Bourret SL (2021) Recovery of freshwater invertebrates in alpine lakes and streams following eradication of nonnative trout with rotenone. J Fish Wildl Manag 12:475–484. 10.3996/JFWM-20-040

[CR124] Schuurman GW et al. (2025) Clarifying the role of resist-accept-direct framework in supporting resource management planning processes. Conserv Biol 39: e70062. 10.1111/cobi.70062 40391403 10.1111/cobi.70062 PMC12309628

[CR125] Schuurman GW et al. (2022) Navigating ecological transformation: Resist–accept–direct as a path to a new resource management paradigm. BioScience 72:16–29. 10.1093/biosci/biab067

[CR126] Schuurman GW, et al. (2020) Resist-accept-direct (RAD)-A framework for the 21st-century natural resource manager. Natural Resource Report. National Park Service, Fort Collins, Colorado. 10.36967/nrr-2283597

[CR127] Scott JM, Loveland T, Gergely K, Strittholt J (2004) National wildlife refuge system: Ecological context and ingegrity. Nat Resour J 44:1041.

[CR128] Sesser AL et al. (2025) Integrating the resist-accept-direct framework into natural resource decision-making process for climate adaptation. Conservation Biology e70116. 10.1111/cobi.70116 10.1111/cobi.70116 40746043

[CR129] Shepard BB, May BE, Urie W (2005) Status and conservation of westslope cutthroat trout within the western United States. North Am J Fish Manag 25:1426–1440. 10.1577/M05-004.1

[CR130] Shultz A et al. (2022) Case study: Applying the resist–accept–direct framework to an Ojibwe Tribe’s relationship with the natural world. Fish Manag Ecol 29:392–408. 10.1111/fme.12568

[CR131] Siikamäki P, Kangas K, Paasivaara A, Schroderus S (2015) Biodiversity attracts visitors to national parks. Biodivers Conserv 24:2521–2534. 10.1007/s10531-015-0941-5

[CR132] Squillace M (2014) Grazing in wilderness areas. Environ Law 2:415–445

[CR133] Stephenson NL, Millar C (2014) Climate change: Wilderness’s greatest challenge. In: Sample VA, Bixler RP (eds) Forest conservation and management in the Anthropocene: Conference proceedings RMRS-P-71. U.S. Forest Service Rocky Mountain Research Station vol 71. p 453-460. https://www.fs.usda.gov/rm/pubs/rmrs_p071/rmrs_p071_453_460.pdf

[CR134] Stoddard JL, et al. (2003) Response of surface water chemistry to the Clean Air Act Amendments of 1990. Environmental Protection Agency. EPA 620/R-03/001. https://nepis.epa.gov

[CR135] Stubbington R, England J, Sarremejane R, Watts G, Wood PJ (2024) The effects of drought on biodiversity in U. K. river ecosystems: Drying rivers in a wet country. WIREs Water 11: e1745. 10.1002/wat2.1745

[CR136] Sudduth EB, Meyer JL, Bernhardt ES (2007) Stream restoration practices in the southeastern United States. Ecol Restor 15:573–583. 10.1111/j.1526-100X.2007.00252.x

[CR137] Sullivan PL, Gaiser EE, Surratt D, Rudnick DT, Davis SE, Sklar FH (2014) Wetland ecosystem response to hydrologic restoration and management: The Everglades and its urban-agricultural boundary (FL, USA). Wetlands 34:1–8. 10.1007/s13157-014-0525-2

[CR138] Sweet LC et al. (2019) Congruence between future distribution models and empirical data for an iconic species at Joshua Tree National Park. Ecosphere 10: e02763. 10.1002/ecs2.2763

[CR139] Syslo JM, Guy CS, Bigelow PE, Doepke PD, Ertel BD, Koel TM (2011) Response of non-native lake trout (Salvelinus namaycush) to 15 years of harvest in Yellowstone Lake, Yellowstone National Park. Can J Fish Aquat Sci 68:2132–2145. 10.1139/f2011-122

[CR140] Thompson LM et al. (2021) Responding to Ecosystem Transformation: Resist, Accept, or Direct?. Fisheries 46:8–21. 10.1002/fsh.10506

[CR141] Underhill JC (1989) The distribution of Minnesota fishes and late Pleistocene glaciation. J Minn Acad Sci 55:32–37

[CR142] U.S. Fish and Wildlife Service (1996) Endangered and threatened wildlife and plants; determination of threatened status for the California red-legged frog *Rana draytonii*. Federal Register 50: 25813–25833 https://www.federalregister.gov/documents/1996/05/23/96-12901/endangered-and-threatened-wildlife-and-plants-determination-of-threatened-status-for-the-california

[CR143] U.S. Fish and Wildlife Service (1999) Endangered and threatened wildlife and plants; determination of threatened status for bull trout in the conterminous United States. Federal Register 64: 58910–58933. https://www.federalregister.gov/documents/1999/11/01/99-28295/endangered-and-threatened-wildlife-and-plants-determination-of-threatened-status-for-bull-trout-in

[CR144] U.S. Fish and Wildlife Service (2002) Endangered and threatened wildlife and plants; listing of the Chiricahua leopard frog (*Rana chiricahuensis*); final rule. Federal Register 67: 40790-40811. https://www.federalregister.gov/documents/2002/06/13/02-14730/endangered-and-threatened-wildlife-and-plants-listing-of-the-chiricahua-leopard-frog-rana

[CR145] U.S. Fish and Wildlife Service (2011a) Endangered and threatened wildlife and plants; endangered status for the Ozark hellbender salamander. vol 76: 61956, Federal Register 76: 61956-61978. https://www.federalregister.gov/documents/2011/10/06/2011-25690/endangered-and-threatened-wildlife-and-plants-endangered-status-for-the-ozark-hellbender-salamander

[CR146] U.S. Fish and Wildlife Service (2011b) Chiricahua leopard frog (*Lithobates* [= *Rana*] *chiricahuensis*) 5-year status review: Summary and evaluation. Albuquerque, New Mexico. https://www.fws.gov/node/260204

[CR147] U.S. Fish and Wildlife Service (2016) 2016 national survey of fishing, hunting and wildlife-associated recreation. https://www.fws.gov/sites/default/files/documents/news-attached-files/nat_survey2016.pdf

[CR148] U.S. Forest Service, Flathead National Forest (2006) South Fork Flathead watershed westlope cutthroat trout conservation program, record of decision. Flathead County, Montana. https://fwp.mt.gov/binaries/content/assets/fwp/conservation/fisheries-management/cutthroat-trout/south-fork-project/forest-service-rod-may-4-2006.pdf. Accessed 17 Decemebr 2024

[CR149] U.S. Forest Service, Lolo National Forest (2024) Decision notice for the terms and conditions for McKinley Lake Dam decommissioning and restoration project. Missoula County, MT. https://www.ci.missoula.mt.us/DocumentCenter/View/71693/DecisionNotice20240422signed

[CR150] U.S. Forest Service, George Washington and Jefferson National Forests (2011) Decision notice and finding of no significant impact for St. Mary’s aquatic mitigation project. Roanoke, WA.

[CR151] Valentim HIL, Feio MJ, Almeida SFP (2024) Fluvial protected areas as a strategy to preserve riverine ecosystems—a review. Biodivers Conserv 33:439–462. 10.1007/s10531-023-02774-w

[CR152] Vredenburg VT (2004) Reversing introduced species effects: Experimental removal of introduced fish leads to rapid recovery of a declining frog. Proc Natl Acad Sci 101:7646–7650. 10.1073/pnas.0402321101 15136741 10.1073/pnas.0402321101 PMC419660

[CR153] Vukomanovic J, Randall J (2021) Research trends in U.S. national parks, the world’s “living laboratories. Conserv Sci Pr 3: e414. 10.1111/csp2.414

[CR154] Wade AA, Theobald DM, Laituri MJ (2011) A multi-scale assessment of local and contextual threats to existing and potential U.S. protected areas. Landsc Urban Plan 101:215–227. 10.1016/j.landurbplan.2011.02.027

[CR155] Ward NK et al. (2023) Reimagining large river management using the Resist–Accept–Direct (RAD) framework in the Upper Mississippi River. Ecol Process 12: 48. 10.1186/s13717-023-00460-x

[CR156] Whiteley AR, Fitzpatrick SW, Funk WC, Tallmon DA (2015) Genetic rescue to the rescue. Trends Ecol Evol 30:42–49. 10.1016/j.tree.2014.10.009 25435267 10.1016/j.tree.2014.10.009

[CR157] Wiley RW (2003) Planting trout in Wyoming high-elevation wilderness waters. Fisheries 28:22–27. 10.1577/1548-8446(2003)28[22 PTIWHW]2.0.CO;2

[CR158] Williams NC, Downey DM (2010) St. Mary’s Acid Mitigation Project: Is it time for another dose of “medicine”? James Madison University, p 1-19

[CR159] Wohl E (2021) Legacy effects of loss of beavers in the continental United States. Environ Res Lett 16:025010. 10.1088/1748-9326/abd34e

[CR160] World Wildlife Fund (2018) Living Planet Report 2018: Aiming higher. Grooten M, Almond REA (eds). Gland, Switzerland. https://www.worldwildlife.org/pages/living-planet-report-2018

[CR161] World Wildlife Fund (2022) Living Planet Report 2022: Building a naturepositive society. Almond REA, Grooten M, Juffe Bignolo D, Peterson T (eds). Gland, Switzerland. https://www.worldwildlife.org/pages/living-planet-report-2022

[CR162] Zellmer SB (2012) Wilderness, water, and climate change. Enviornmental Law 42:313

[CR163] Zellmer SB (2014) Wilderness management in national parks and wildlife refuges. Environ Law 44:497

